# Factors influencing the posterior tibial slope after medial opening-wedge high tibial osteotomy

**DOI:** 10.3389/fbioe.2025.1525542

**Published:** 2025-03-21

**Authors:** Junwei Li, Qingqing Yang, Min Zhang, Jie Yao, Bolun Liu, Yichao Luan, Yunlin Chen, Chaohua Fang, Cheng-Kung Cheng

**Affiliations:** ^1^ Key Laboratory of Biomechanics and Mechanobiology, Ministry of Education, Beijing Advanced Innovation Center for Biomedical Engineering, School of Biological Science and Medical Engineering, Beihang University, Beijing, China; ^2^ School of Biomedical Engineering and Engineering Research Center for Digital Medicine of the Ministry of Education, Shanghai Jiao Tong University, Shanghai, China; ^3^ Department of Spine Surgery, No. 6 Hospital of Ningbo, Ningbo, Zhejiang, China; ^4^ Department of Sports Medicine, Shanghai General Hospital, Shanghai Jiao Tong University School of Medicine, Shanghai, China; ^5^ Department of Orthopedic, Bengbu Hospital of Shanghai General Hospital (The Second Affiliated Hospital of Bengbu Medical University), Bengbu, Anhui, China

**Keywords:** high tibial osteotomy, lower limb alignment, posterior tibial slope, ratio between anterior and posterior, hinge axis

## Abstract

**Introduction:**

Medial Opening-wedge High Tibial Osteotomy (HTO) is an effective treatment for medial compartment osteoarthritis and knee varus in relatively young and active patients. While it can effectively correct lower limb alignment in the coronal plane, it may also affect the posterior tibial slope (PTS) in the sagittal plane. However, the factors influencing PTS and methods for maintaining PTS stability remain controversial.

**Methods:**

A lower limb geometric model was constructed based on the CT data from a patient with medial knee osteoarthritis and varus knee. Multiple models were developed to simulate various conditions: seven different medial cortex inclinations of the proximal tibia (–15°–15°), seven coronal plane inclinations of the central osteotomy plane (–15°–15°), seven sagittal plane inclinations of the hinge axis (–15°–15°), seven hinge axis heights (–7 mm–7 mm), and seven hinge axis inclinations in the axial plane (–15°–15°). Changes in the ratio between anterior and posterior opening gap (RAPOG) and PTS were analyzed.

**Results:**

The medial cortex inclination of the proximal tibia, coronal plane inclination of the central osteotomy plane, inclination of the sagittal plane of the hinge axis, and height of the hinge axis did not alter the PTS; however, these factors did affect RAPOG, with increased values leading to decrease in RAPOG. The ranges of RAPOG for these factors were 76.37%–54.83%, 68.91%–60.94%, 68.04%–64.08%, and 70.38%–62.61%, respectively. However, the hinge axis inclination on the axial plane affects PTS, for inclinations of –15°, –10°, –5°, 0°, 5°, 10°, and 15°, the PTS decreased 2.48°, 1.83°, 0.98°, 0°, –0.97°, –1.82°, and –2.53°, respectively. To maintain a constant PTS, RAPOG should be readjusted to 65.13%, 66.01%, 66.27%, 65.76%, 65.03%, 65.15%, and 65.57%, respectively.

**Discussion:**

The inclination of the hinge axis in the axial plane affects PTS, as its value increases, PTS also increases. To maintain a constant PTS, RAPOG should be readjusted. Understanding these relationships is essential for optimizing surgical techniques to minimize unintended changes in PTS.

## 1 Introduction

Medial opening-wedge high tibial osteotomy (HTO) is a mature and effective treatment for relatively young and active patients with medial compartment osteoarthritis and knee varus (Meding et al., 2011; Valenzuela et al., 2013; Portner, 2014; Elyasi et al., 2021; Bai et al., 2023). It is used to correct coronal malalignment and transfer the initial lower limb from the overloaded and worn medial compartment to the lateral compartment, thereby reducing pressure in the medial compartment, alleviating knee joint pain, delaying medial compartment cartilage wear, and improving knee function (d’Entremont et al., 2014; Portner, 2014; Chung et al., 2021; Elyasi et al., 2021). However, it can simultaneously increase the posterior tibial slope (PTS) by 2°–5° in the sagittal plane (Wang et al., 2009; Moon et al., 2015; Ozel et al., 2017; Kim et al., 2019; Kaya et al., 2020; Teng et al., 2021; Yoon et al., 2023). The alteration of PTS can lead to biomechanical changes in the knee joint. The increase in PTS causes the tibial to move anteriorly relative to the femur, often triggering degenerative changes in the anterior cruciate ligament (Jiang et al., 1994; Kim et al., 2019). This also results in degenerative changes in the femoral and tibial articular cartilage (Rodner et al., 2006; Savarese et al., 2011; Liukkonen et al., 2023) and the risk of fractures at the hinge axis (Nakamura et al., 2015; Nakamura et al., 2017). Therefore, identifying the risk factors causing changes in PTS and how to maintain PTS unchanged are crucial aspects of patient treatment.

Usually, the surgeon adjusts the PTS by changing the ratio between anterior and posterior opening gaps (RAPOG). The geometric shape of the proximal tibia is characterized by an angle of approximately 45° formed between the anteromedial and lateral cortexes of the tibial (Noyes et al., 2005). Noyes et al. (2005) found that the PTS was unchanged when the RAPOG was 50%. Song et al. (2007) used navigation-assisted HTO and reported that the PTS was unchanged when the RAPOG was 67%. Using postoperative radiograph images after HTO, Yoon et al. (2023) found that the PTS was unchanged when the RAPOG was 70%. These results highlight the inconsistency in the reported RAPOG values required to maintain constant PTS.

In addition to RAPOG, other factors that affect PTS after HTO include the hinge position, hinge axis direction, and angle between the hinge and the central osteotomy plane (Noyes et al., 2005; Slope, 2009; Chung et al., 2021; Teng et al., 2021). On the coronal plane, the transverse osteotomy barely alters PTS compared to the oblique osteotomy (Slope, 2009). On the sagittal plane, Moon et al. (2021) demonstrated that a forward-tilted hinge axis results in decreased PTS, while a backward-tilted hinge axis leads to increased PTS. On the axial plane, the PTS increased when the hinge axis was rotated externally. Conversely, the PTS decreased (Wang et al., 2009; Moon et al., 2015; Eliasberg et al., 2021). In summary, several factors affect the PTS during HTO. However, few studies have investigated how to adjust the RAPOG value to maintain a constant PTS when the above mentioned factors changed, and there is a lack of consensus on the reported results.

This study aimed to reconstruct a three-dimensional geometric model of the lower limb based on a patient’s preoperative computed tomography (CT) data, establish a standard HTO model, and analyze: (1) Do the medial cortex inclination of the proximal tibia, the coronal plane inclination of the central osteotomy plane, the sagittal plane inclination of the hinge axis, the height of the hinge axis, and the hinge axis on the axial plane did affect the PTS? (2) How can the stability of PTS be maintained? We hypothesized that, firstly, the medial cortex inclination of the proximal tibia, the coronal plane inclination of the central osteotomy plane, the sagittal plane inclination of the hinge axis, and the height of the hinge axis did not affect the PTS, but did affect the RAPOG. Secondly, it was hypothesized that the inclination of the hinge axis on the axial plane noticeably affects the PTS.

## 2 Materials and methods

**FIGURE 1:**
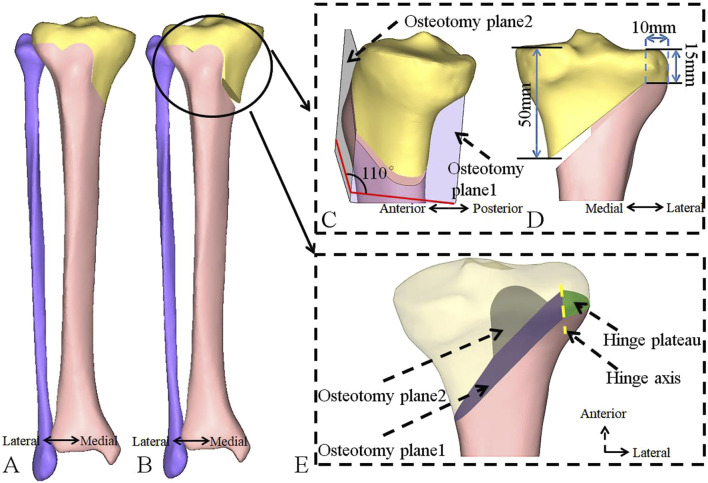
Establishment of a standardized research model for HTO using CT data. **(A)** Before the osteotomy. **(B)** After the osteotomy. **(C)** Enlarged sagittal view of the osteotomy plane. **(D)** Enlarged coronal view of the osteotomy plane. **(E)** Enlarged coronal view of the osteotomy plane, with the purple area representing osteotomy plane 1 (the central osteotomy plane), the gray area representing osteotomy plane 2 (superior osteotomy plane), the yellow line representing hinge axis and the green area representing the hinge plateau. HTO, high tibial osteotomy; CT, computed tomography.

**FIGURE 2:**
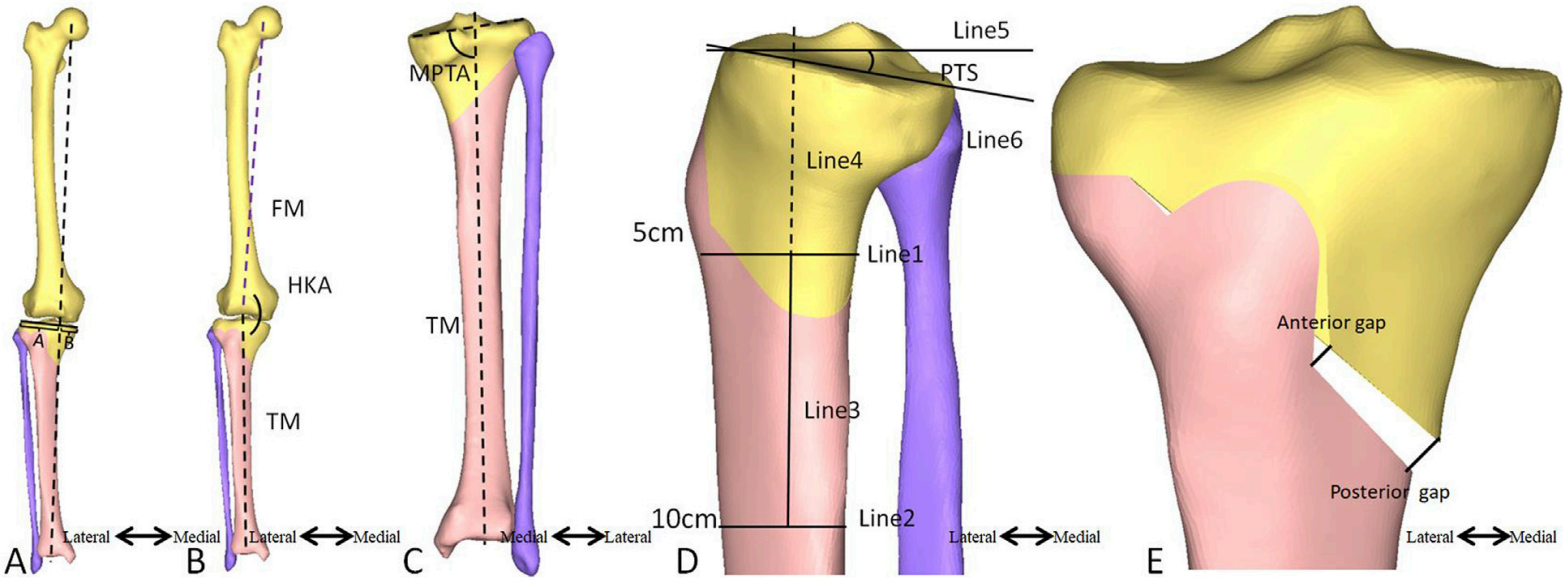
Measurement of the lower limb alignment, HKA, MPTA, PTS, anterior opening gap, and posterior opening gaps. **(A)** The lower limb alignment is considered to be aligned when a vertical line running from the center of the femoral head passes through the center of the articular plane of the talus and links the medial edge and lateral edge of the tibia. The lower limb alignment ratio is B/(A + B). **(B)** The HKA angle is formed between the first line connecting the center of the femoral head to the center of the tibia and the second line connecting the center of the tibia to the center of the articular plane of the talus. **(C)** The MPTA angle is formed between the first tibial anatomical axis and the medial and lateral connecting lines of the second tibial plateau. **(D)** The anterior and posterior tibial cortex points on the distal end of the joint line at 5 cm and 15 cm, respectively, link the line connecting the anterior and posterior cortical points (Line 1, Line 2). Then, the midpoint of the connecting line is established. The proximal anatomical axis connects the two points (Line 3). A vertical line (Line 5) to the proximal tibial extension line (Line 4) is plotted, and a line (Line 6) between the leading edge and the posterior edge of the medial tibial plateau can be defined. The angle between Line 5 and Line 6 is the PTS angle. **(E)** The anterior opening gap is measured at the medial edge of the coronal osteotomy site, and the posterior opening gap is measured at the tibia’s most prominent posterior medial edge. HKA, hip-knee-ankle; MPTA, medial proximal tibial angle; PTS, posterior tibial slope; FM, femoral mechanical axis; TM, tibial mechanical axis.

This study was conducted under the approval of the institutional review board. A CT scan was performed on the femur, tibia, and fibula of a 62-year-old female patient, 165 cm tall, weighing 58.3 kg. The CT data was imported into Mimics 20.0 (Materialise, Inc., Belgium) to reconstruct the geometric morphology of the bones. Geomagic Studio 14.0 (Raindrop Geomagic, Inc., Morrisville, NC) was used for surface smoothing and feature extraction. The obtained three-dimensional geometric model was imported into Hypermesh (Altair Engineering, Inc., United States) to simulate a HTO procedure. The preoperative lower limb alignment, Hip-Knee-Ankle (HKA) angle, Medial proximal tibial angle (MPTA), and PTS, as well as postoperative lower limb alignment, HKA angle, MPTA, PTS, anterior opening gap, and posterior opening gap, were measured (Figures 1, 2) (Song et al., 2007; Wang et al., 2009; Green et al., 2020; Chung et al., 2021; Erquicia et al., 2022). The HTO orthopedic target was MPTA = 90.00° (Smith et al., 2013). Tibial osteotomy was performed according to the standard operating procedure for HTO, as follows:

1) The central osteotomy plane is located 5.0 cm below the medial articular surface of the tibial plateau, directed towards below the lateral articular surface of the tibia plateau by 1.5 cm, and positioned 1.0 cm within the inner side of the lateral cortical surface of the tibia (Figures 1C, D).
2) The superior osteotomy start at the posterior edge of the patellar tendon insertion point on the tibial tuberosity and forms an angle of 110° with the central osteotomy plane (Figure 1C).
3) The osteotomy gap was opened evenly, and MPTA was corrected to 90.00°.
4) The posterior inclination of the medial tibial plateau, as well as the anterior and posterior opening gap of the central osteotomy plane, was measured.
5) If the change in PTS was ≥1° after orthopedic treatment, the anterior opening gap and posterior opening gap of the central osteotomy plane were adjusted to ensure that the postoperative PTS was equal to the preoperative PTS.

**FIGURE 3:**
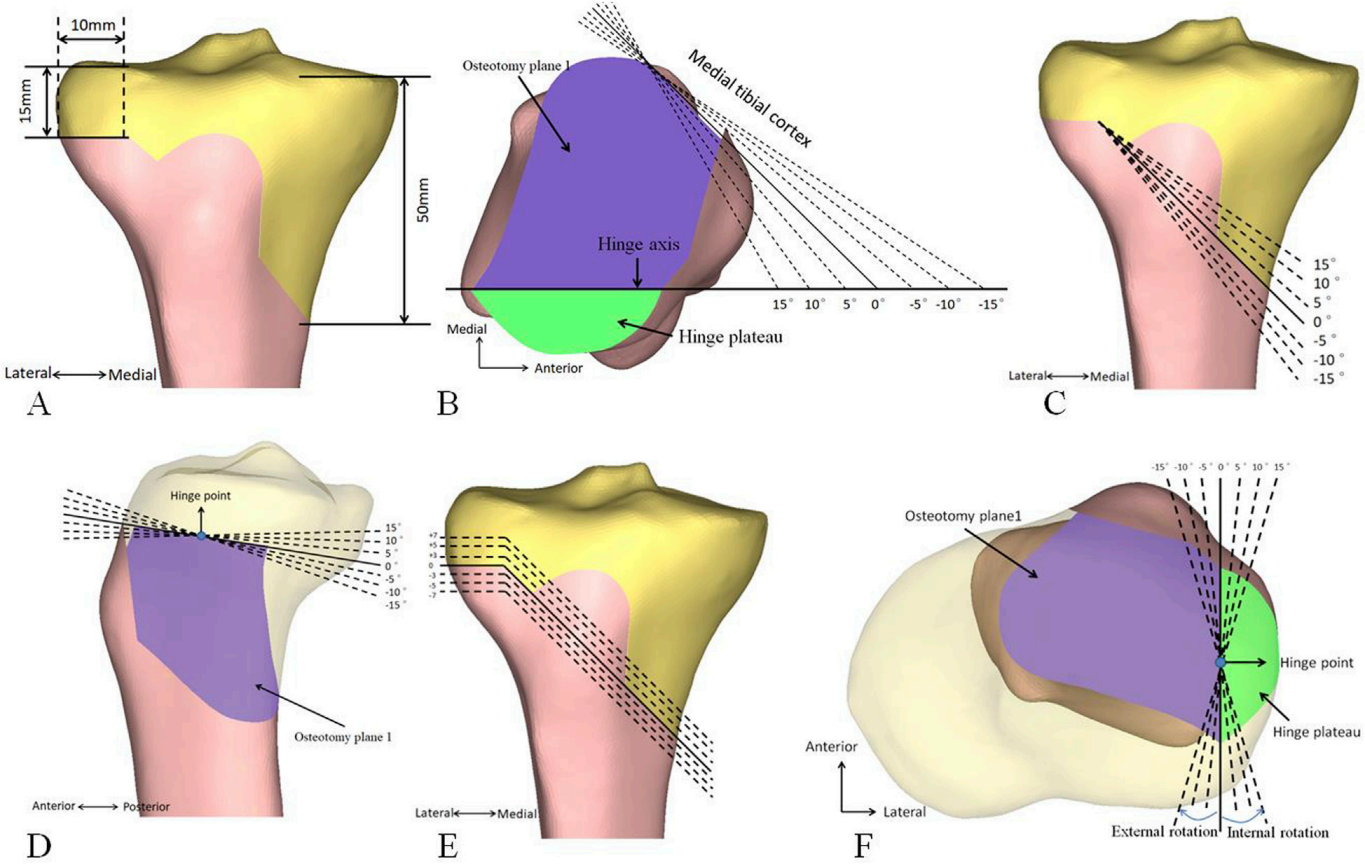
Standard HTO and other osteotomies. **(A)** Standard osteotomy. **(B)** Medial cortex inclination of the proximal tibia. **(C)** Coronal inclination of the central osteotomy plane. **(D)** The sagittal inclination of the hinge axis. **(E)** Height of the hinge axis. **(F)** The inclination of the hinge axis on the axial plane. HTO, high tibial osteotomy.

Using a standard osteotomy as the baseline (Figure 3A), seven models were developed for each of the following: (i) different inclination angles for the medial cortex of the proximal tibia (−15°, −10°, −5°, 0°, 5°, 10°, 15°), with a positive angle indicating the inclination is towards the lateral tibial plateau (Figure 3B), (ii) different inclination angle of the central osteotomy plane on the coronal plane (−15°, −10°, −5°, 0°, 5°, 10°, 15°), with a positive angle indicating the inclination is towards the proximal tibia (Figure 3C), (iii) different inclination angle of the hinge axis on the sagittal plane (−15°, −10°, −5°, 0°, 5°, 10°, 15°), with a positive angle indicating a forward tilt (Figure 3D), (iv) different heights of the hinge axis (−7 mm, −5 mm, −3 mm, 0 mm, 3 mm, 5 mm, and 7 mm), with a positive value towards the proximal tibia (Figure 3E), and (v) different inclinations of the hinge axis on the axial plane (−15°, −10°, −5°, 0°, 5°, 10°, 15°), with internal rotation indicated by a negative angle (Figure 3F). The postoperative HKA angle, MPTA, PTS, and anterior and posterior opening gap were measured, and the RAPOG was calculated.

## 3 Results

### 3.1 Standard HTO

**FIGURE 4:**
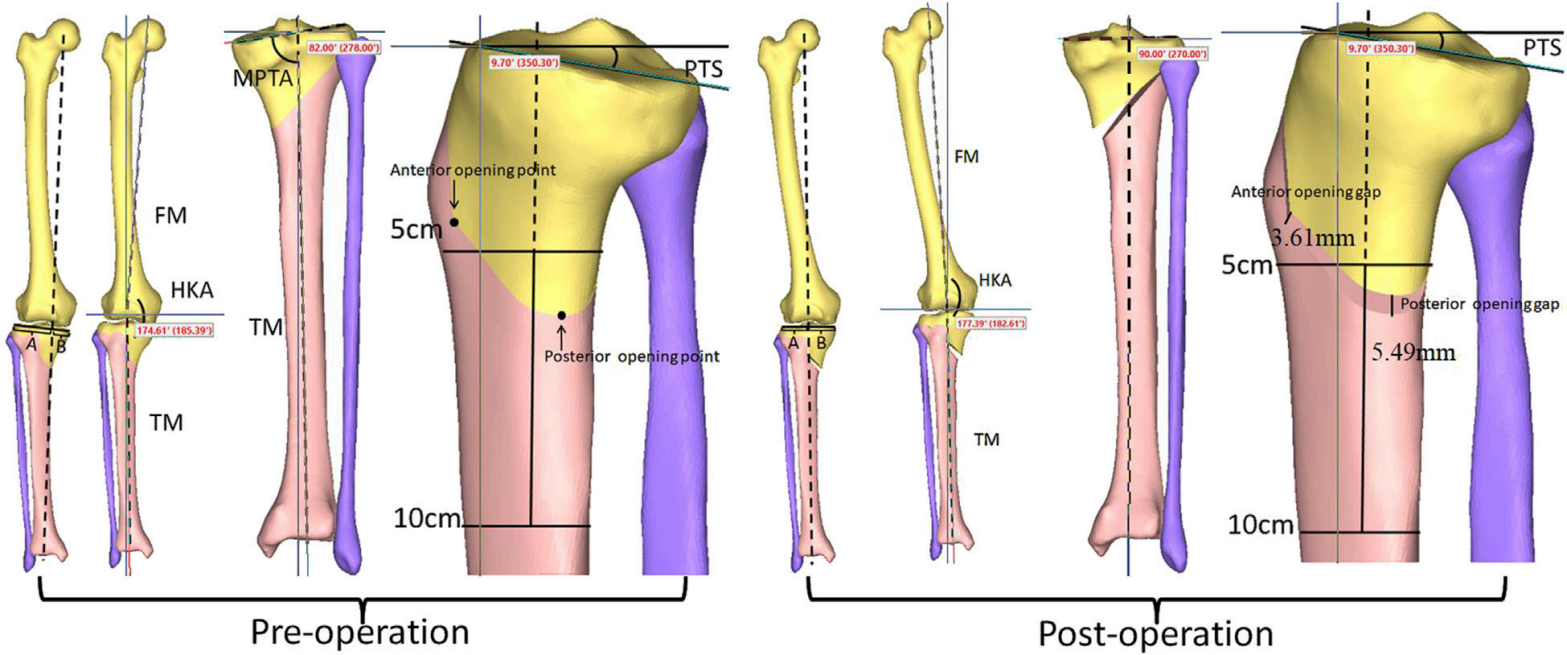
Changes in lower limb alignment, HKA angle, MPTA, and PTS before and after standard osteotomy. HKA, hip-knee-ankle; MPTA, medial proximal tibial angle; PTS, posterior tibial slope; FM, femoral mechanical axis; TM, tibial mechanical axis.

**FIGURE 5:**
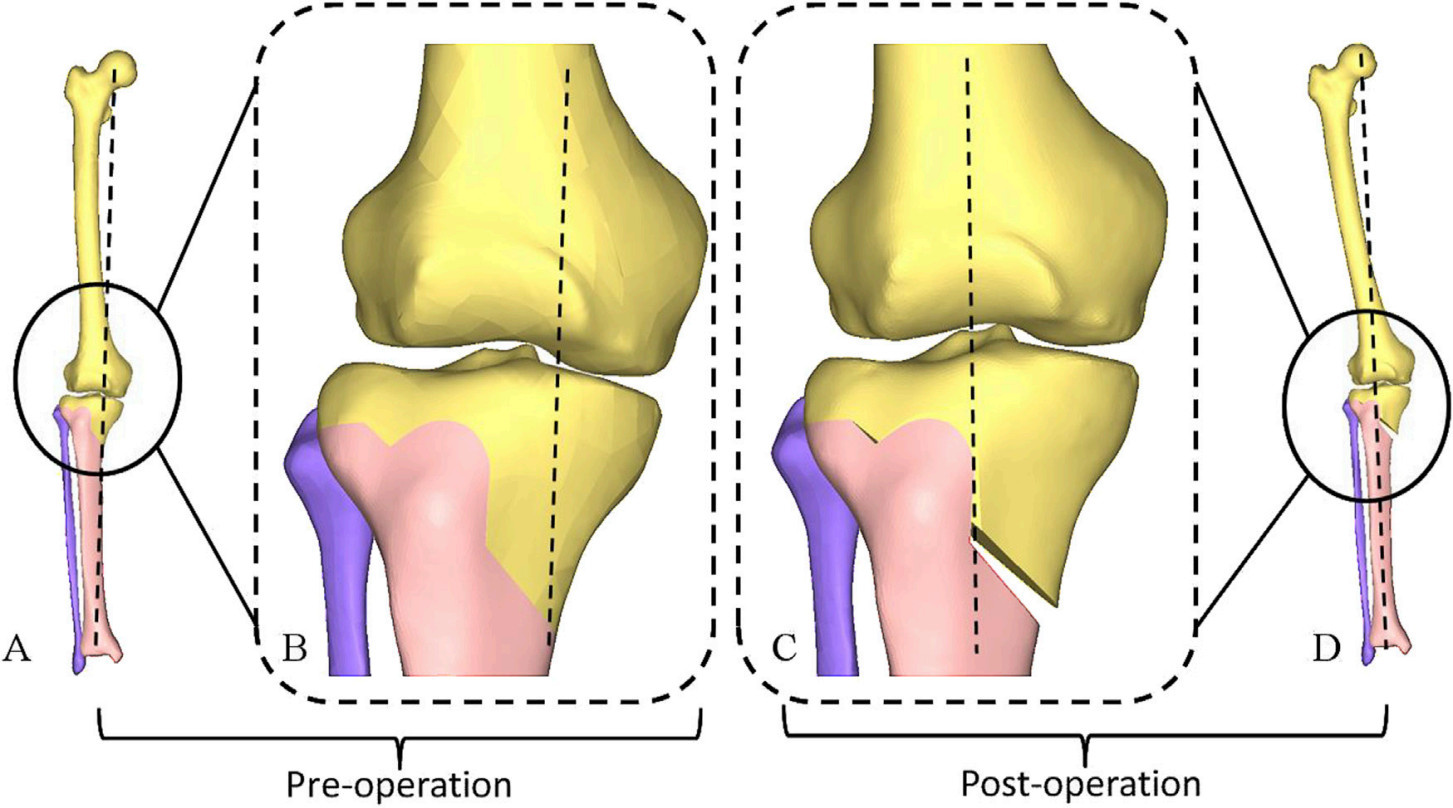
Changes in lower limb alignment Pre- and Post-Operatively. **(A)** Before the osteotomy. **(B)** Enlarged Coronal view in **(A)**. **(C)** Enlarged Coronal view in **(D)**. **(D)** After the osteotomy.

The preoperative HKA angle, MPTA, and PTS were recorded as 174.61°, 82.00°, and 9.70°, respectively. When a standard HTO was simulated and the MPTA adjusted to 90.00°, the PTS was unchanged (Figure 4). The lower limb alignment transferred from the medial compartment of the knee joint to the lateral compartment, with an HKA of 182.61° (Figures 4, 5). The anterior opening gap of 3.61 mm, the posterior opening gap of 5.49 mm, and the RAPOG of 65.76% (Figure 4).

### 3.2 Which factors affect the PTS and RAPOG?

**FIGURE 6:**
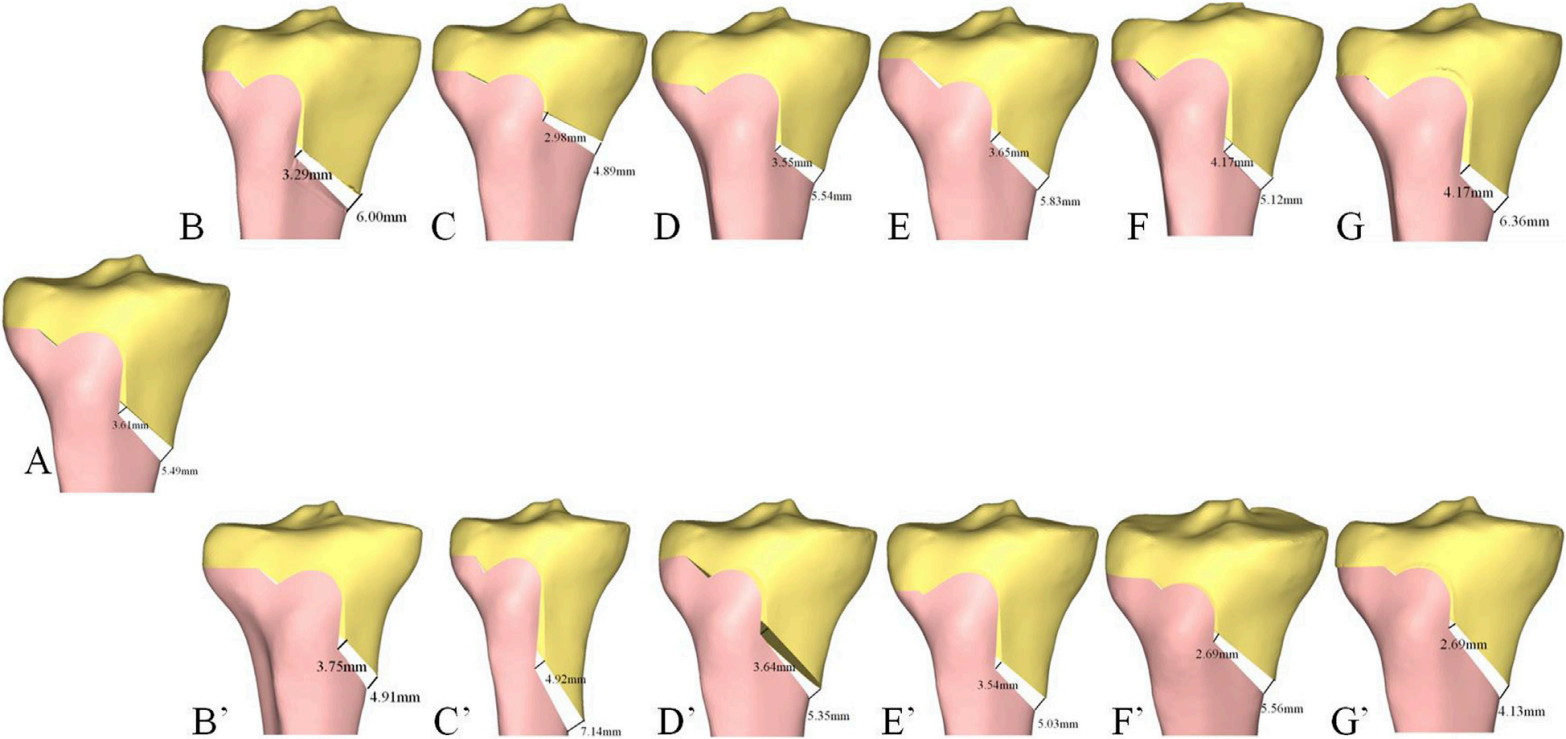
Changes in RAPOG after HTO under the influence of different factors. **(A)** Standard osteotomy for HTO. **(B)** Medial cortex inclination of the proximal tibia (15°). (B’) Medial cortex inclination of the proximal tibia (-15°). **(C)** Coronal plane inclination of the central osteotomy plane (15°). (C’) Coronal plane inclination of the central osteotomy plane (−15°). **(D)** The Sagittal plane inclination of the hinge axis (15°). (D’) The Sagittal plane inclination of the hinge axis (−15°). **(E)** Height of the hinge axis (7 mm). (E’) Height of the hinge axis (-7 mm). **(F)** The inclination of the hinge axis on the axial plane (15°). (F’) The inclination of the hinge axis on the axial plane (−15°). **(G)** Adjust PTS-Inclination of the hinge axis on the axial plane (15°). (G’) Adjust PTS-Inclination of the hinge axis on the axial plane (−15°).

When the osteotomy gap was opened evenly, the medial cortex inclination of the proximal tibia, the coronal plane inclination of the central osteotomy plane, the sagittal plane inclination of the hinge axis, and the height of the hinge axis did not change the postoperative PTS. However, there was a noticeable change in the RAPOG. In Figure 6 (B-B’, C-C’, D-D’ E-E’), we show the changes in the opening gap at two extreme angles and positions under each factor change to demonstrate the resulting changes in RAPOG. As the values of these factors increased, RAPOG decreased (Table 1). Specifically, for the medial cortex inclination of the proximal tibia, the RAPOG was 76.37%, 72.91%, 70.84%, 65.76%, 62.39%, 59.06%, and 54.83%; for the coronal plane inclination of the central osteotomy plane, the RAPOG was 68.91%, 68.72%, 67.76%, 65.76%, 64.35%, 62.98%, and 60.94%; for the sagittal plane inclination of the hinge axis, the RAPOG was 68.04%, 67.10%, 66.30%, 65.76%, 65.15%, 64.67% and 64.08%; for the height of the hinge axis, the RAPOG was 70.38%, 67.75%, 66.32%, 65.76%, 65.29%, 64.85% and 62.61% (Table 1).

**TABLE 1:** The effect of the different factors on the RAPOG and PTS.

	Postoperative-preoperative PTS (°)	Anterior opening gap (mm)	Posterior opening gap (mm)		RAPOG
Medial cortex inclination of the proximal tibia (°)					
15	0	3.29		6.00	54.83%
10	0	3.39		5.74	59.06%
5	0	3.50		5.61	62.39%
0	0	3.61		5.49	65.76%
−5	0	3.62		5.11	70.84%
−10	0	3.66		5.02	72.91%
−15	0	3.75		4.91	76.37%
Coronal plane inclination of the central osteotomy plane (°)	0				
15	0	2.98		4.89	60.94%
10	0	3.13		4.97	62.98%
5	0	3.34		5.19	64.35%
0	0	3.61		5.49	65.76%
−5	0	3.93		5.80	67.76%
−10	0	4.35		6.33	68.72%
−15	0	4.92		7.14	68.91%
The sagittal plane inclination of the hinge axis (°)					
15	0	3.55	5.54		64.08%
10	0	3.57	5.52		64.67%
5	0	3.59	5.51		65.15%
0	0	3.61	5.49		65.76%
−5	0	3.62	5.46		66.30%
−10	0	3.63	5.41		67.10%
−15	0	3.64	5.35		68.04%
Height of the hinge axis (mm)					
7	0	3.65	5.83		62.61%
5	0	3.64	5.61		64.85%
3	0	3.63	5.56		65.29%
0	0	3.61	5.49		65.76%
−3	0	3.58	5.40		66.32%
−5	0	3.55	5.24		67.75%
−7	0	3.54	5.03		70.38%

### 3.3 How can the stability of PTS be maintained?

**FIGURE 7:**
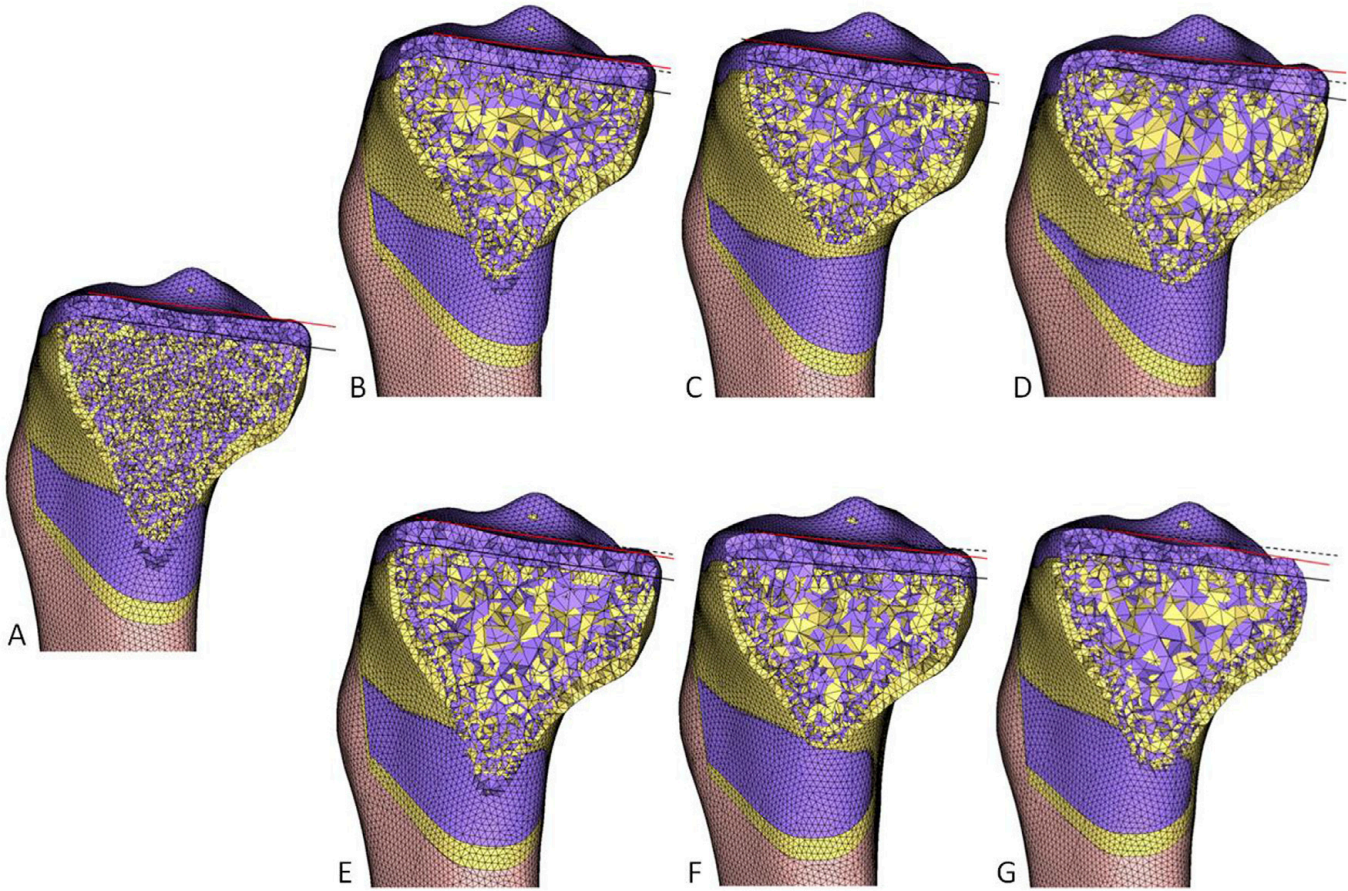
The effect of the hinge axis inclination on the axial plane on PTS. Yellow represents the pre-operative, and purple represents the post-operative. The black solid line connects the leading edge to the trailing edge of the medial tibial plateau. The black dashed line runs parallel to the black solid line, and the red solid line connects the leading edge to the trailing edge of the medial tibial plateau after surgery. The angle between the red solid line and the black dashed line represents the change in PTS. **(A)** standard osteotomy. **(B)** −5°. **(C)** −10°. **(D)** −15°. **(E)** 5°. **(F)** 10°. **(G)** 15°. PTS, posterior tibial slope.

The inclination of the hinge axis on the axial plane noticeably affected postoperative PTS. PTS increased (external rotation) as the values increased (Figure 7). The difference between the postoperative and preoperative PTS was −2.48°, −1.83°, −0.98°, 0°, 0.97°, 1.82° and 2.53°, respectively. There was also a noticeable change in RAPOG, which was 48.38%, 54.79%, 60.62%, 65.76%, 70.93%, 76.14%, and 81.45%, respectively. To maintain the postoperative PTS unchanged, the posterior opening gap had to be decreased or increased during internal or external rotation of the hinge axis, respectively, and the RAPOG had to be adjusted to 65.13%, 66.01%, 66.27%, 65.76%, 65.03%, 65.15%, and 65.57%, respectively (Table 2).

**TABLE 2:** The effect of the inclination of the hinge axis on the axial plane on the RAPOG and PTS.

The inclination of the hinge axis on the axial plane (°)	Postoperative-preoperative PTS (°)	Anterior opening gap (mm)	Posterior opening gap (mm)	RAPOG	Adjusted posterior opening gap (mm)	RAPOG
15	2.53	4.17	5.12	81.45%	6.36	65.57%
10	1.82	4.02	5.28	76.14%	6.17	65.15%
5	0.97	3.83	5.40	70.93%	5.89	65.03%
0	0	3.61	5.49	65.76%	5.49	65.76%
−5	−0.98	3.34	5.51	60.62%	5.04	66.27%
−10	−1.83	3.03	5.53	54.79%	4.59	66.01%
−15	−2.48	2.69	5.56	48.38%	4.13	65.13%

## 4 Discussion

This study demonstrated that, in addition to maintaining the PTS, standard HTO restored the MPTA to 90.00° and adjusted the HKA angle from 174.61° preoperative to 182.61° postoperative. This adjustment effectively transferred the lower limb alignment from medial to lateral, thus reducing the pressure in the medial compartment (Figures 4, 5). For Changes in RAPOG and PTS due to surgical parameters, the results showed that changes in the medial cortex inclination of the proximal tibia, coronal plane inclination of the central osteotomy plane, sagittal plane inclination of the hinge axis, and height of the hinge axis did not alter the PTS but considerably affected the RAPOG, which was consistent with first hypothesis. As the values of the factors above increased, the RAPOG gradually decreased, ranging from 76.37% to 54.83%, 68.91%–60.94%, 68.04%–64.08%, and 70.38%–62.61%, respectively. However, the inclination of the hinge axis on the axial plane notably changed the postoperative PTS (Table 2; Figure 7), which was consistent with our second hypothesis. When the hinge axis changed by −15°, −10°, −5°, 0°, 5°, 10°, and 15° on the axial plane, the PTS changed by −2.48°, −1.83°, −0.98°, 0°, 0.97°, 1.82°, and 2.53°, respectively. Meanwhile, the RAPOG values were 48.38%, 54.79%, 60.62%, 65.76%, 70.93%, 76.14%, and 81.45%, respectively. To maintain the same PTS, the RAPOG of the primary osteotomy needed to be adjusted to 65.13%, 66.01%, 66.27%, 65.76%, 65.03%, 65.15%, and 65.57%, respectively.

Recognized factors affecting PTS include RAPOG, geometrical morphology of the proximal tibia, and position and angle of the hinge axis (Lobenhoffer and Agneskirchner, 2003; Noyes et al., 2005; Song et al., 2007; Slope, 2009; Jo et al., 2018; Teng et al., 2021; Yoon et al., 2023). Compared to clinical or cadaveric experimental studies, the research study method had noticeable advantages in its quantitative nature, high accuracy, and repeatability (Kuriyama et al., 2019; Chung et al., 2021; Yoon et al., 2023). Regarding the inclination of the hinge axis on the axial plane, previous studies (Moon et al., 2015; Teng et al., 2021) found that the hinge axis rotating externally can significantly increase the postoperative PTS. Our study was consistent with the above conclusions, demonstrating that the rotation of the hinge axis internally or externally on the axial plane can noticeably decrease or increase PTS. A 10° external rotation of the hinge axis resulted in a 1.82° increase in PTS, while a 10° internal rotation decreased PTS by 1.83°. To maintain a constant PTS, the RAPOG must be readjusted. However, adjusting the anterior and posterior opening gap may increase the stress at the hinge axis and cause fracture (Wright et al., 2005; Martin et al., 2014; Nakamura et al., 2015; Ogawa et al., 2017; Weng et al., 2017). Studies have shown that drilling holes along the hinge axis can effectively reduce the stress at the hinge axis, thereby decreasing the occurrence of fractures (Boström et al., 2021). On the sagittal plane, Chung et al. (2021) found that tilting the hinge axis posteriorly on the sagittal plane increased the PTS, while an anterior tilt decreased the PTS. On the contrary, Moon et al. (2021) stated that the posterior angle of the hinge axis on the sagittal plane decreased the PTS, while an anterior tilt increased the PTS. Teng et al. (2021) reported that the inclination of the hinge axis on the sagittal plane did not change the PTS, which was consistent with our results (Teng et al., 2021). On the coronal plane, a related study found that the PTS increased from preoperative 7.7° ± 4.6° to postoperative 10.7° ± 3.8° after oblique osteotomy, while the PTS hardly changed when a transverse osteotomy was used (Slope, 2009). Jo et al. (2018) performed HTO on 16 cadaveric knee joints and compared the effect of the height of the hinge axis on the PTS in the coronal plane. They found that a higher hinge axis could reduce the increase in the PTS. However, our results indicated that the height of the hinge axis and the distance from the starting point of the central osteotomy plane to the joint line did not affect the PTS. Therefore, to avoid inadvertent effects on the kinematics of the knee joint, close attention needs to be paid to prevent the hinge axis in the axial plane from undergoing internal or external rotation during HTO. Additionally, in cases where the treatment aims to intentionally adjust the PTS, such as for osteoarthritis with anteroposterior instability, the PTS can be controlled by varying the hinge axis inclination in the axial plane.

Regarding the RAPOG, Lobenhoffer and Agneskirchner (2003) showed that RAPOG should be 1:1 to prevent a change in the post-operative PTS. However, Lee et al. (2016) suggested that the RAPOG should be 63% to maintain a constant PTS. Song et al. (2007) used navigation-assisted HTO and reported that the PTS was unchanged when the RAPOG was 67%. Yoon et al. (2023) found that the PTS was unchanged when the RAPOG was 70%. The results of this study were closed with song’s study (Song et al., 2007). It was found that in standard HTO, maintaining the RAPOG at 65.76% can maintain the PTS unchanged.

We recommend that during the HTO procedure, surgeons should pay attention to the patient’s medial cortex inclination of the proximal tibia, the coronal plane inclination of the central osteotomy plane, the inclination of the sagittal plane of the hinge axis, the height of the hinge axis and the inclination of the hinge axis on the axial plane, to determine RAPOG for each patient, rather than applying the same RAPOG to all patients. Furthermore, surgeons should pay particular attention to the inclination of the hinge axis on the axial plane to prevent postoperative change in PTS.

The present study has several limitations. Firstly, as our study is based on a theoretical three-dimensional surgical simulation, it does not account for the influence of other factors, such as soft tissue, or surgical techniques on PTS. Secondly, this study only considered the influence of individual factors on RAPOG and PTS. Although this study focused on the effects of changes in individual factors on PTS and RAPOG, it still provides valuable insights into the factors influencing changes in PTS following HTO and the RAPOG required to maintain a stable PTS. Future research should consider the impact of multiple factors on RAPOG and PTS. Thirdly, the study simulated HTO using CT data from one patient.

## 5 Conclusion

In HTO, the hinge axis on the axial plane considerably influences the PTS: internal rotation of the hinge axis decreases PTS, while external rotation increases it. Conversely, factors such as the medial cortex inclination of the proximal tibia, the coronal plane inclination of the central osteotomy plane, the sagittal plane inclination of the hinge axis, and the height of the hinge axis did not alter PTS, but did alter RAPOG. Therefore, when altering the osteotomy position and angle in HTO, it is essential to pay attention to RAPOG to maintain a stable PTS.

## Data Availability

The original contributions presented in the study are included in the article/supplementary material, further inquiries can be directed to the corresponding authors.
